# Biochemical and Physiological Studies on the Effects of Senescence Leaves of *Populus deltoides* on *Triticum vulgare*

**DOI:** 10.1155/2014/126051

**Published:** 2014-12-24

**Authors:** Tejinder Pal Khaket, Viney Kumar, Jasbir Singh, Suman Dhanda

**Affiliations:** Department of Biochemistry, Kurukshetra University, Kurukshetra, Haryana 136119, India

## Abstract

*Triticum vulgare* (Wheat) based products are the major dietary source of food in developing countries. In India, it grows in association with boundary plantations of *Populus deltoids* (poplar). During winter, poplar enters in dormancy which cause a heavy leaf fall at the time of wheat seed germination. Large number of poplar senescence leaves may adversely affect the wheat. Therefore, the present study was performed to examine the effect of senescence poplar leaves on wheat germ and some other biochemical parameters. Seed's germination rate was determined by measuring root and shoot lengths, percent germination, germination index, and inhibition percentage. Biochemical parameters, namely, pigment, carbohydrate, protein, and phenol content, were estimated. Activities of catalase and polyphenol oxidase which are stress marker enzymes were also measured. Results revealed that germination and other biochemical parameters of wheat were severely affected by senescence poplar leaves even at very low concentration. So, intercropping of poplar along with wheat may be chosen carefully as wheat is the major dietary staple.

## 1. Introduction

Wheat (*Triticum* spp.) is a cereal grain that belongs to family* Poaceae*. In 2010, world production of wheat was 651 million tons, making it the third most-produced cereal after maize (844 million tons) and rice (672 million tons) [1]. Wheat has relatively higher protein content than other major cereals, namely, maize (corn) and rice. Globally, it is leading source of plant protein in human food. Currently, it is next to rice but leads maize as maize is more extensively used in animal feed.

Wheat is an important winter crop. In India it is being grown in association with the boundary plantation of* Populus deltoides*. The genus Populus belongs to family* Salicaceae* that includes more than hundred species distributed in temperate and subtropical regions.* Populus deltoides* based agroforestry system is one of the alternate land use systems to obtain biological production on a sustainable basis in irrigated agroecosystem. Owing to its fast growth, deciduous nature, marketing acceptability, and successful intercropping, poplar cultivation is common among the farmers as a viable alternative to wheat-paddy rotation in Northwestern states of India. Poplar is advantageous to rabi crop as it enters in dormancy during winter and therefore enhances food production and thus economics returns of the growers. This species have been grown as a boundary or block plantation that improves the physicochemical properties of soil through the addition of organic matter in the soil [2, 3] and provides an alternate source of income and employment [4, 5].

However, some adverse effects have also been reported by various workers [6–8]. Fallen leaves of poplar purposed to affect companion crop by decreasing the availability of nutrients and/or light to developing seedlings and changing C : N ratio of the soil [3]. During winter, senescence leaves of poplar result in heavy load of poplar phytochemicals which may affect physiological and biochemical properties of intercropped wheat. In the light of the above mentioned facts, the present study was conducted to evaluate the effect of senescence leaves of* Populus deltoides* on the seed germination and other biochemical parameters of wheat.

## 2. Material and Methods

### 2.1. Materials

Fine chemicals and reagents were purchased from Sigma-Aldrich chemicals, Bangalore Genei, Fischer, Rankem, Himedia, Ranbaxy, India. All chemicals were of analytical grade.

### 2.2. Collection of Plant Material

Seeds of* Tricitum vulgare *HD 2733 were obtained from IARI, regional station, Karnal (India). Senescence leaves of poplar (*Populus deltoides*) were collected from agricultural fields of Kurukshetra, Haryana, India.

### 2.3. Extract Preparation of Senescence Leaves of Poplar

Collected leaves were dried at room temperature for 8–10 days. Then 10% aqueous extract was prepared and diluted to different concentrations (0.001%, 0.01%, 0.1%, 1%, and 10%).

### 2.4. Seed Germination

Wheat seeds were surface sterilised with 0.1% (w/v) mercuric chloride for 1 min and 75% ethanol for 5 min. Subsequently, seeds were thoroughly rinsed with an excess of distilled water and imbibed overnight and then sewn up to one week with daily irrigation of different working concentrations of senescence poplar leaf extract for test and water for control.

### 2.5. Measurement of Seed Germination

The wheat seed germination rate was determined by measuring root and shoot lengths, percent germination, and germination index [9]:

(1) Germination  index  =Germination  %×Root  length  of  the  testRoot  length  of  control.

Inhibition percentage due to senescence poplar leaves was calculated using the following modified equation [10]:

(2) $$\begin{array}{c} \text{Inhibition}\;\;\text{percentage}=\frac{\text{Treated}-\text{Control}}{\text{Control}}\times 100. \end{array}$$

### 2.6. Estimation of Chlorophyll and Carotenoids

Chlorophyll and carotenoid contents of wheat leaves were determined spectrophotometrically [11]. Absorbance of acetone extract of wheat leaves was recorded at 663, 647, and 470 nm for chlorophyll a, chlorophyll b, and carotenoids, respectively. The contents were expressed as mg chlorophyll a and chlorophyll b and carotenoids g^−1^ fresh weight.

- Total  chlorophyll  content  (per g tissue weight) = 20.2 × (*A*_647_) + 8.02 × (*A*_663_):
   Chl a  content = 12.7 × *A*_663_ − 2.69 × *A*_647_  (*μ*g/mL  in  extract);
   Chl b  content = 22.9 × *A*_647_ − 4.68 × *A*_663_  (*μ*g/mL  in  extract).
- Carotenoid  content  (*μ*g/g) = (*A*_470_ × *V* × 10^4^)/(*A*_1_^1%^ × *P*):
   
*A* = Absorbance, *V* = total extract volume in mL, *P* = sample weight in grams, and *A*_1_^1%^ cm = 2592 (*β*-carotene extinction coefficient).

### 2.7. Total Soluble Sugar Content

Total soluble sugar was quantified by anthrone method of Yemm and Willis [12].

### 2.8. Estimation of Total Phenol

The total phenol content of wheat leaves was estimated by Folin-Ciocalteu method [13]. Wheat leaves extract (10%) was prepared in Tris-HCl (pH-7.4) buffer and centrifuged at 3000 rpm for 15 min. 46 mL of distilled water and 1 mL Folin-Ciocalteu reagent were added to the supernatant and mixed vigorously. After 3 min, 3 mL of 2% Na_2_CO_3_ was added and the mixture was allowed to stand for 2 h with intermittent shaking and absorbance was measured at 760 nm. Total phenol content was estimated with reference to a standard curve of catechol.

### 2.9. Protein Estimation

Total protein content was estimated by the method of Lowry et al. [14] using standard curve of BSA as reference.

### 2.10. Protein Profiling by SDS-PAGE

Protein profiling was studied on 10% SDS-PAGE [15]. Protein bands were detected by Coomassie Brilliant Blue staining.

### 2.11. Proline Content

Proline content was measured using ninhydrin reagent [16]. Fresh sample (300 mg each) was homogenized in 10 mL of 3% aqueous sulfosalicylic acid and centrifuged at 9000 ×g for 15 min. Supernatant (2 mL) was mixed with equal volume of acetic acid and ninhydrin and incubated for 1 h at 100°C. The reaction was terminated in ice bath. The product was extracted with 4 mL of toluene by vortexing for 20 s and absorbance was read at 520 nm against toluene. Proline content was measured using standard curve of catechol.

### 2.12. Extraction of Enzymes

The frozen sample was homogenized with pestle and mortar in prechilled 50 mM sodium phosphate buffer (pH 7.0) containing 5 mM *β*-mercaptoethanol and 1 mM EDTA. The homogenate was centrifuged at 12,000 ×g for 15 min at 4°C. The supernatant was used as a source of enzymes.

### 2.13. Assay of Antioxidant Enzymes

#### 2.13.1. Catalase (CAT, E.C. 1.11.1.6)

Catalase activity was assayed by following a decline in observance of H_2_O_2_ at 240 NM (*ε* = 39.4 M^−1^ cm^−1^) [17]. The reaction mixture consisted of 50 *μ*L of enzyme extract in 50 mM sodium phosphate buffer (pH 7.0). The reaction was started by addition of H_2_O_2_ to a final concentration of 10 mM, and its consumption was measured up to 2 min with 30 seconds time interval. One unit of catalase activity is defined as the amount of enzyme that catalyzes the oxidation of 1 mole of H_2_O_2_ per minute under the assay conditions. Catalase activity (Units/mg) was calculated using following formula:

(3) $$\begin{array}{c} \text{Units}/\text{mg}=\frac{\Delta A_{240}\;\text{nm}/\min\times 1000}{43.6\times \text{mg}\;\;\text{enzyme}/\text{mL}\;\;\text{reaction}\;\;\text{mixture}}. \end{array}$$

#### 2.13.2. Polyphenol Oxidase (EC 1.14.18.1)

Polyphenol oxidase was assayed in 3 mL of reaction mixture containing 50 mM sodium phosphate, 0.17 mM of L-DOPA, 0.07 mM L-ascorbic acid, 0.065 mM of EDTA, and 0.1 mL of wheat leaves extract. After incubation at 25°C change in absorbance at 265 nm was recorded for approximately 5 min [18]. PPO activity was calculated using the following formula:

(4) Units/mg  enzyme  =(A265 nm/min⁡  Test−A265 nm/min⁡  Blank)0.001(mg  enzyme/3)

0.001 = the change in A_265_ nm/minute per unit polyphenol oxidase at pH 6.5 at 25°C in a 3 mL reaction mixture. One unit of PPO activity was defined as the amount of enzyme that caused the change in *A*_265_ nm of 0.001 per minute at pH 6.5 at 25°C in a 3 mL reaction mix containing L-3,4-dihydroxyphenylalanine and L-ascorbic acid.

### 2.14. Statistical Methods

All results were analysed using descriptive statistical techniques such as mean standard deviation. One-way ANOVA was employed to test the significance and *P* < 0.05 was considered statistically significant. Tukey's multiple-range test was performed for multiple-parameter analysis. All statistical analysis was performed by GraphPad Prism 5.0.

## 3. Results and Discussion

Effect of aqueous extract of poplar senescence leaves on wheat germination was studied with respect to wheat's physiological and biochemical parameters.

### 3.1. Physiological Effects

Germination studies revealed a decrease in germination index with increase in concentration of poplar senescence leaves (PSL). Germination index was inhibited up to 60% at 10% PSL concentration (Table 1). As roots are involved in nutrient absorption, studies of the effect of PSL extracts on roots of crop under investigation become compulsory. PSL also significantly (*P* < 0.05) affected shoot and root lengths of germinating HD 2733VSM variety. Significant negative correlation existed between PSL extract and root and shoot lengths. PSL showed strong inhibitory effect toward root length as compared to shoot length (60% and 51.06%, resp.). The decreased germination and root and shoot length of wheat at higher PSL concentration might be due to excess of inhibitory phytochemicals. Allelopathic effect of poplar on seed germination and radical growth of wheat were also reported by Melkania [19]. Catechol and benzoic acid inhibitors of poplar leaves were supposed to be the major cause of their inhibitory effect [8, 20].

Reduction in germination, plant height, and biomass at 30 and 60 days after sowing of some winter season crops such as* T. aestivum, L. culinaris, Phaseolus mungo, Avena sativa, T. alexandrinum,* and* Brassica juncea* was observed when these crops were grown under* P. deltoides* as compared to treeless fields [7]. The reduced yield of agriculture crops was due to allelopathic interference of phytotoxin phenolics from leaves and litter of poplar which accumulated in soil. Wheat grain yield also decreased in a sheltered field of* Populus deltoids* mainly because of phytotoxic interference of phenolics released into soil [21–23].

### 3.2. Biochemical Changes

#### 3.2.1. Photosynthetic Pigments

**Figure 1:**
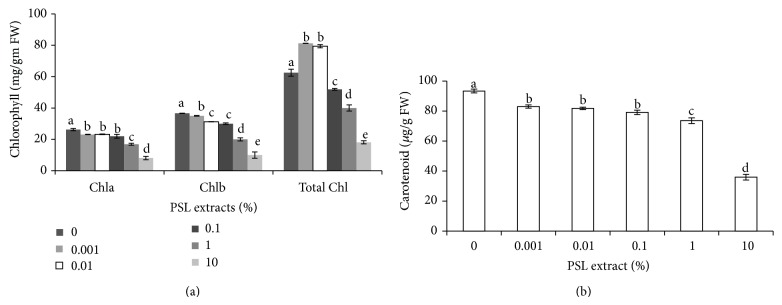
Effect of PSL extract on chlorophyll content (a) and carotenoid content (b) of wheat. Error bars represents Mean ± S.D of three different experiments. Data were subjected to one-way ANOVA analysis and differences among treatments were determined by Tukey's test. Different letters mean statistical differences at *P* < 0.05.

Chl a, Chl b, and total chlorophyll content were also measured for wheat in the presence of the poplar leaves extract. ANOVA results showed significant reduction (*P* < 0.05) in chlorophyll content after treatment with PSLs at each concentration (Figure 1(a)). Initially, chlorophyll content was significantly (*P* < 0.05) increased up to 0.01% PSL extract but decreased thereafter and decrease was more pronounced with an increase in PSL concentration. Chlorophyll a and chlorophyll b content decreased approximately to 70% at 10% PSL concentration. Chl b was found more sensitive to phytochemicals and decreased significantly up to 67% as compared to Chl a (>70%). It is also supported by earlier observations by Singh and Rao [24]. Chlorophylls are photosynthetic pigments and reduction in their content will also be associated with reduced rates of photosynthesis. On the other hand, carotenoids serve as light absorbing pigments which play a vital role in photosynthesis. Carotenoid content declined by 21.3% to 61.56% at 1 to 10% PSL concentration (Figure 1(b)). High concentration of PSL might had severe due to high levels of phytochemicals and free radical damage.

Decline in chlorophyll and carotenoids is also associated with senescence. Breakdown of chlorophylls may be one of the earliest symptoms of senescence. The chlorophyll a/b ratio declined with the advancement of senescence [25, 26], probably due to nonsynchronous dismantling of lamellae and grana thylakoids and the asymmetrical distribution of photosystems between them. Carotenoids are lost at a much lower rate than chlorophylls [27]. In senescing barley leaves, chlorophyll b reduction seems to be the first and obligatory step of chlorophyll b breakdown that is carried out by chlorophyll(ide) b reductase, a thylakoidal enzymatic activity that peaks earlier (day 2) than chlorophyllase (day 4) during dark-induced leaf senescence [28].

### 3.3. Other Stress Factors

Plants subjected to environmental stress accumulate a wide range of soluble nitrogenous and other compounds in higher concentration that play a specific role in plant defense.

### 3.4. Total Soluble Sugar

**Figure 2:**
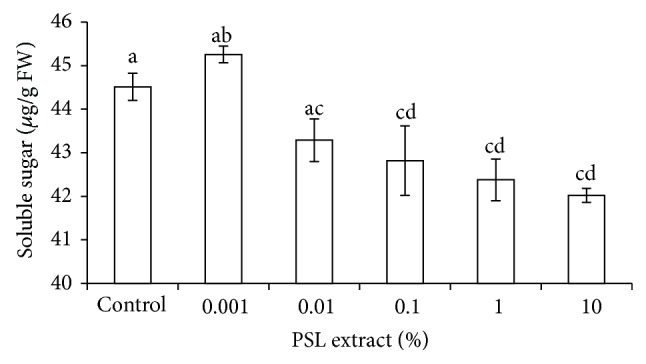
Effect of PSL extract on sugar contents of wheat. Error bars represents Mean ± S.D of three different experiments. Data were subjected to one-way ANOVA analysis and differences among treatments were determined by Tukey's test. Different letters mean statistical differences at *P* < 0.05.

Total sugar content changed slightly in the presence of PSL extracts (Figure 2). Initially, it increased at 0.001% PSL concentration but decreased thereafter from 0.01 to 10% (5.87% reduction). Result of one-way ANOVA revealed that soluble sugar content of wheat at 0.001 and 0.01% PSL did not significantly differ compared to control. In addition, soluble sugar of wheat in the presence of 0.01% onward was also not significantly differed. Significant difference between treated and untreated wheat supported sensitivity of carbohydrate metabolism to stress and allelochemicals because they interfere with biosynthetic processes [24].

### 3.5. Total Phenol Content

**Figure 3:**
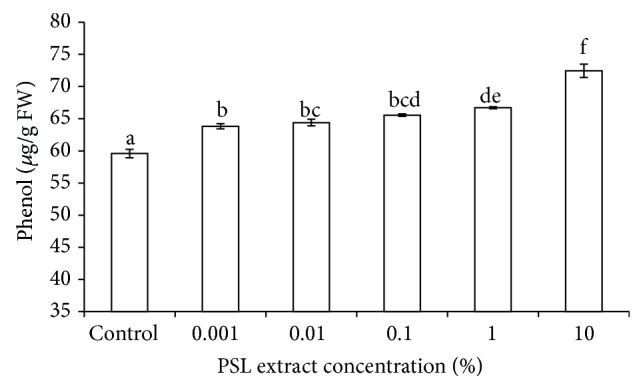
Effect of PSL extract on phenol content of wheat. Error bars represent Mean ± S.D of three different experiments. Data were subjected to one-way ANOVA analysis and differences among treatments were determined by Tukey's test. Different letters mean statistical differences at *P* < 0.05.

Phenol content increased significantly with an increase in PSL extract concentration. Increase in phenol content at 10% PSL concentration was significant up to 95% level of significance, as predicted by one-way ANOVA (Figure 3). Increased phenol content was found to be responsible for reducing seedling growth in various abiotic stresses [29]. Phenolic content increased in allelopathic conditions also. Phenolic compounds interfere with phosphorylation pathway or decreased synthesis of carbohydrates, proteins, nucleic acids, and secondary metabolites and also interfere with cell division, mineral uptake, and some other biosynthetic processes [29–31].

### 3.6. Protein Content

**Figure 4:**
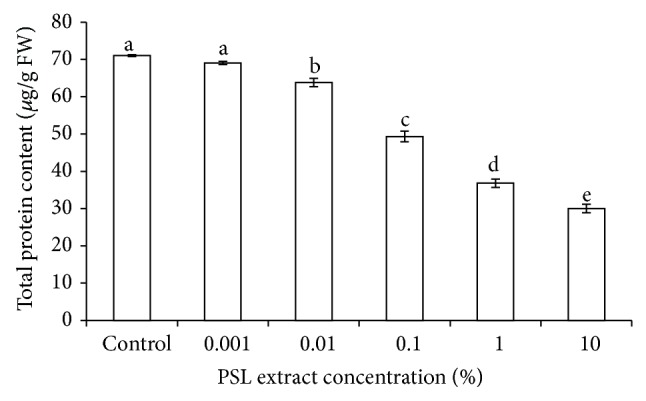
Effect of PSL extract on total protein content of wheat. Error bars represent Mean ± S.D of three different experiments. Data were subjected to one-way ANOVA analysis and differences among treatments were determined by Tukey's test. Different letters mean statistical differences at *P* < 0.05.

Environmental stress is generally detrimental to plant growth and adversely affect protein synthesis and hydrolysis in plants which cause an imbalance in the level of protein [32–35]. One-way ANOVA analysis results revealed that the total protein content of wheat was significantly (*P* < 0.05) reduced in presence of PSL (Figure 4). Protein content started declining at 0.001% PSL extract concentration and reduction was more severe with an increase in PSL extract concentration. Protein content reduced by 58% at 10% PSL extract. Decrease in total protein content may be due to increase in phenol content because many phenolic acids such as Ferulic acid, chlorogenic acid, vanillic acid, and p-coumaric acid are known to reduce the incorporation of certain amino acid into proteins and thus reduce the rate of protein synthesis [36–38]. Moreover, in salt- and water-stressed plant parts, the protein content also decreases owing to decreased rate of protein synthesis and increased rate of proteolysis [32, 39, 40]. Studies conducted so far indicated that stressful conditions alter the protein metabolism in plants and new stress specific proteins are synthesized in all different types of environmental stresses, such as salinity, drought, heat, chilling, anaerobiosis, pathogenesis, wounding, heavy metal toxicity, and gaseous pollutants [41].

**Figure 5:**
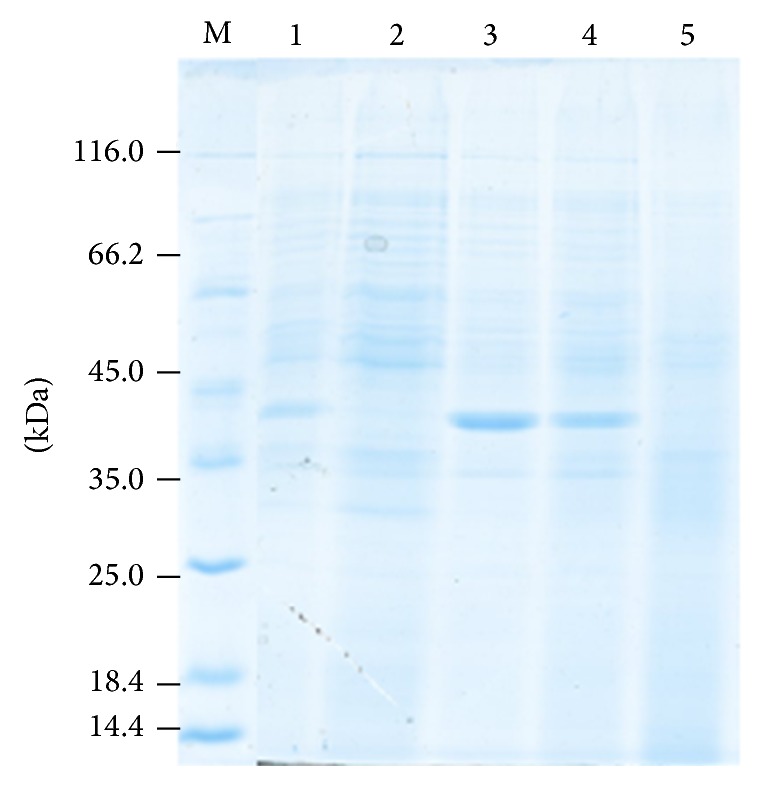
SDS-PAGE (10%) of heat proteins. Lane 1 has control and lanes 2–5 have wheat leaves treated with 0.001, 0.01, 0.1, and 10% poplar senescence leaves. M stands for marker lane.

**Figure 6:**
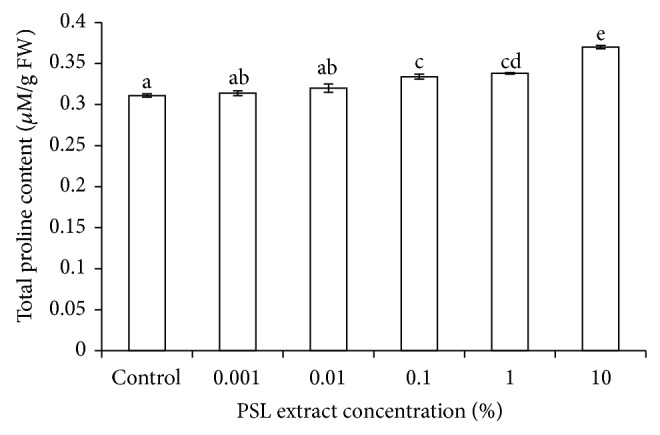
Effect of PSL extract on total proline content of wheat. Error bars represent Mean ± S.D of three different experiments. Data were subjected to one-way ANOVA analysis and differences among treatments were determined by Tukey's test. Different letters mean statistical differences at *P* < 0.05.

After quantification PSL treated proteins were analyzed by SDS-PAGE (Figure 5). With the increase in PSL extract concentrations, intensity of protein bands decreased and at 10% almost all protein bands disappeared or if present they are of very low intensity. It might be due to severe toxicity of heavy phytochemicals at increased extract concentration. Two polypeptides of 41.31 and 39.61 kDa appeared with the high band intensity on treatment with 0.001% and 0.01–0.1% extracts (Figure 6). These proteins might be involved in stress management of plants.

### 3.7. Proline Content

Abiotic stresses induced accumulation of many compounds such as ascorbate, glutathione, *α*-tocopherol, betaine, proline, and other amino acids, quaternary ammonium compounds, polyamines, sucrose, polyols (mannitol, sorbitol, and pinitol), and oligosaccharides in the affected plant [42].

Proline content of wheat leaves increased from 1.2 to 20.1% with an increase in PSL concentration from 0.001 to 10%, respectively (Figure 6). Proline content is positively correlated up to a significant level with stress severity which may be either due to inhibition of protein oxidation or due to breakdown of protein from its precursors [43]. Proline is also involved in intracellular osmotic adjustment [44, 45].

### 3.8. Antioxidant Enzymes

Reactive oxygen species (ROS) are generated under stress conditions and antioxidant enzymes protect the cell structures against ROS [45]. Phytotoxins are proposed to work through the production of ROS and relative oxidative stress [46]. ROS are also known to trigger induction and expression of defense enzymes [45]. Production of soluble enzymatic and nonenzymatic antioxidants is one of the major protective means of the plant against ROS. Chemical defense against free radicals include compounds that are strong reducing agent such as glutathione, phenols, flavonoids, and polyamines [47]. Enzymatic defenses against free radicals include superoxide dismutase, catalase, peroxidase, phenol oxidase, and ascorbic acid oxidase [45, 48].

Catalase and polyphenol oxidase (PPO) activities increased approximately by 28% and 16% at 10% PSL extract concentration, respectively. The increased activity of both enzymes might be due to production of reactive oxygen or nitrogen species such as hydrogen peroxide and polyphenols, which further generate more free radicals. Catalase scavenges hydrogen peroxide with water and dioxygen production (nontoxic compounds) [45]. Increased polyphenol activity was purposed to be a possible tolerance mechanism for plants under stress condition such as freezing and salt stress in* C. angustifolia* [49]. Different types of allelochemicals also stimulated many antioxidant enzyme activities in response to high level of free radicals in* Lycopersicon esculentum* [50].

## 4. Conclusion

Intercropping of compatible crops is the need of hour to fulfill the increased dietary demand of an increasing population. Intercropping of poplar with wheat is beneficial for farmers to some extent. But senescence leaves of poplar adversely affect the seedling growth and other biochemical properties of wheat. Therefore, required improvement and care should be taken in using intercropping approach of poplar and wheat, so that only beneficial properties are utilized and harmful properties are avoided.

## Figures and Tables

**Table 1:** Root, shoot length, and germination index of wheat seeds in the presence of PSL extract.

Concentration (%)	Root length (cm)	Shoot length (cm)	Germination index (%)	Inhibition percentage
Control	5 ± 0.22^a^	9.4 ± 0.6^a^	100	0
0.001	4.5 ± 0.17^ab^	7.4 ± 0.45^b^	90	10
0.01	4.1 ± 0.09^bc^	6.5 ± 0.19^bc^	82	18
0.1	4.0 ± 0.31^bcd^	6.2 ± 0.11^bc^	80	20
1	3.5 ± 0.13^cd^	5.3 ± 0.51^cd^	70	30
10	2 ± 0.11^e^	4.6 ± 0.28^d^	40	60

Values are Mean ± S.D of three different experiments. Data were subjected to one-way ANOVA analysis and differences among treatments were determined by Tukey's test. Different letters mean statistical differences at *P* < 0.05.
